# Association of the Ratio of Glycoalbumin to Hemoglobin A1c With Season Change and the COVID-19 Pandemic in Patients With Type 2 Diabetes Mellitus

**DOI:** 10.7759/cureus.64444

**Published:** 2024-07-13

**Authors:** Kohzo Takebayashi, Mototaka Yamauchi, Kenji Hara, Takafumi Tsuchiya, Koshi Hashimoto

**Affiliations:** 1 Department of Diabetes, Endocrinology and Hematology, Dokkyo Medical University Saitama Medical Center, Koshigaya, JPN

**Keywords:** ga, type 2 diabetes, covid-19, seasonal variation, ga/hba1c

## Abstract

Aim: The main purpose of the current study was to investigate the effect of season change and the influence of the COVID-19 pandemic on the ratio of glycoalbumin to hemoglobin A1c (GA/HbA1c) in patients with type 2 diabetes.

Patients and methods: A total of 267 patients in whom both HbA1c and GA were measured at baseline were included in this retrospective study. GA/HbA1c was investigated for three years, 2018, 2019, and 2020 (COVID-19 pandemic period).

Results: The mean values for GA/HbA1c per year in 2018, 2019, and 2020 were 2.64±0.35, 2.61±0.35, 2.64±0.39, respectively. There were no significant differences in GA/HbA1c during these years. There was a tendency toward seasonal variation in GA/HbA1c (i.e., higher in summer or autumn and lower in spring or winter).

Conclusion: In patients with type 2 diabetes, GA/HbA1c tended to show seasonal variation, which was not influenced by the COVID-19 pandemic.

## Introduction

Good glycemic control is important in the prevention of the progression of diabetic complications such as microangiopathies (nephropathy, neuropathy, and retinopathy) and macroangiopathies including ischemic heart disease and stroke [1,2]. Hemoglobin A1c (HbA1c) is a product of non-enzymatic glycation in the N terminus of the β chain of hemoglobin [3]. This is considered to represent the average plasma glucose levels for the preceding two to three months [3]. Therefore, HbA1c is now an established marker of diagnosis, long-term glycemic control, and the risk of the progression of complications of diabetes [3,4]. On the other hand, glycemic fluctuation or postprandial hyperglycemia itself also causes diabetic complications possibly via increased oxidative stress [5-9].

Glycoalbumin (GA), another marker of glycemic control, reflects the preceding two to four weeks [10], based on the relatively short half-life time of albumin (approximate 17 days) [11] and more rapid glycation time compared with hemoglobin [12]. Therefore, GA serves as a marker of glycemic control over shorter periods compared with HbA1c. In addition, GA may be useful as a marker of glucose fluctuation or postprandial hyperglycemia [13-15]. Furthermore, the ratio of GA to HbA1c (GA/HbA1c) can reflect glucose fluctuation or postprandial hyperglycemia and is useful as an easily measured marker for these conditions [15-18]. It has been reported that GA/HbA1c is associated with diabetic retinopathy [19] and neuropathy [20], and with progression of atherosclerosis, as evaluated by intimal-medial complex thickness of the carotid artery [21]. GA/HbA1c may also be associated with cognitive impairment [22] or hippocampal atrophy [23] and the risk of Alzheimer's disease [24]. Notably, GA/HbA1c has been used to predict the progression of COVID-19 from mild to severe disease in patients with type 2 diabetes [25] and was found to be related to all-cause and cardiovascular mortality irrespective of the existence of diabetes in a large cohort in the United States [26]. The results of these clinical studies suggest that GA/HbA1c may also serve as a predictive factor of various complications, including those related to diabetes.

However, whether GA/HbA1c has seasonal variation as HbA1c (higher in the cooler season and lower in the warmer season) [27-29], which is not the case with GA [29,30], remains unknown. This may be important when GA/HbA1c is used as a marker of various complications. Therefore, in the current study, we investigated the seasonal variations of GA/HbA1c in patients with type 2 diabetes by reanalyzing the data from our previous retrospective study [29]. In addition, we studied the influence of the COVID-19 pandemic on GA/HbA1c. Furthermore, the effect of season change and the influence of the COVID-19 pandemic on the strength of correlation evaluated by its coefficient between HbA1c and GA were also investigated.

## Materials and methods

From the 290 patients who had registered in a previous retrospective study [29], 267 patients in whom both HbA1c and GA were measured at baseline were included in this study. This study was performed in our hospital located in Koshigaya City, Japan. The residential areas of a very high percentage of the patients were Koshigaya City and the surrounding districts. A total of 68 patients had been treated with insulin, and 137, 119, and 103 patients had received dipeptidyl peptidase (DPP)-4 inhibitors (DPP4-Is), sulfonylureas and glinides, and metformin, respectively. In addition, 67, 19, 18, and 18 patients had taken alpha-glucosidase inhibitors (alpha GIs), glucagon-like peptide-1 receptor agonists (GLP-1 RAs), pioglitazone, sodium-glucose cotransporter 2 inhibitors (SGLT2-Is), respectively. In therapies, most patients were receiving combination therapy. The patients were all Japanese.

Detailed methods have been described in a previous study [29]. In brief, outpatients with type 2 diabetes who had received regular treatment for glycemic control for more than three years (visits from earlier periods to March 2018) and visited the hospital from January 2021 to March 2021 were registered based on a retrospective evaluation of the electronic medical records.

The intervals between each visit were generally two to three months (range one to three months). The years 2018, 2019, and 2020 were defined, respectively, from March 2018 to February 2019, March 2019 to February 2020, and March 2020 to February 2021. The 2020 year was also defined as the period of the COVID-19 pandemic. The seasons of spring, summer, autumn, and winter were defined, respectively, as March to May, June to August, September to November, and December to February. The measurement methods for HbA1c and GA have been described in our previous study [29].

Ethical considerations

The Institutional Ethics Committee at Dokkyo Medical University Saitama Medical Center approved the study with a six-month opt-out period (approval no.: 21014, approval day: April 26, 2021).

Statistical methods

Differences in GA/HbA1c among the four seasons in 2018, 2019, and 2020 and among the three years were assessed using repeated measures analysis of variance (ANOVA) with a post hoc Holm's test. In this analysis, the GA/HbA1c for each season was calculated as the mean of the GA/HbA1c measured at the visit points (one to three points) during the three months for each season, and these were only calculated in patients in whom both HbA1c and GA were measured throughout the four seasons for each year (n =241 in 2018, n =241 in 2019, and n =224 in 2020). Comparisons of these variables among the four seasons were performed for each year (i.e., n=4 for each year), but not for the three years taken together (i.e., n =12 as a total of three years).

Correlations between HbA1c and GA were examined using Pearson's correlation analysis; the composition of the patients examined in each month was somewhat different because of the visit interval of one to three months. The mean correlation coefficient between HbA1c and GA in the seasons or the whole year for each year was measured as the mean of those of the three months in the season (i.e., n=3, for each season) or 12 months in each year (i.e., n =12, for each year). Thus, the differences in the correlation coefficients between HbA1c and GA among the four seasons in each year and over the three years were assessed using ANOVA, but not repeated measures ANOVA, with a post hoc Holm's test, as the patient composition for each interval was not completely the same. All statistical analyses were performed using BellCurve software (Social Survey Research Information Co., Ltd., Tokyo, Japan) for Excel (Microsoft Corporation, Redmond, Washington, United States). A p-value of less than 0.05 was estimated as indicating statistical significance (two-sided).

## Results

The clinical characteristics and laboratory data for the 267 patients at baseline (first visit after March 2018) are summarized in Table 1.

**Table 1:** Clinical features and laboratory data of patients with type 2 diabetes. Data are expressed as mean ± standard deviation. In therapies, most patients were receiving combination therapy. No.: number of patients; BMI: body mass index; HbA1c: hemoglobin A1c; GA: glycoalbumin; eGFR: estimated glomerular filtration rate; DPP4-I: dipeptidyl peptidase 4 inhibitors; AGI: alpha-glucosidase inhibitors; SGLT2-I: sodium-glucose cotransporter 2 inhibitors; GLP-1RA: glucagon-like peptide-1 receptor agonists

Parameter	Value
No. (male/female)	267 (154/113)
Age (year)	70.2±10.9
Duration of diabetes (number of patients in respective classifications)	
0-4 years	11
5-9 years	71
10-14 years	75
≥15 years	110
BMI (kg/m2)	24.6±4.7
HbA1c (%)	7.4±1.1
GA (%)	19.2±4.4
GA/HbA1c	2.60±0.36
eGFR (ml/min/1.73 m2)	63.6±18.4
Diabetes and lipid therapy (number of patients treated with respective drugs)	
Sulfonylureas and glinides	119
Metformin	103
Pioglitazone	18
AGI	67
DPP4-I	137
SGLT2-I	18
GLP-1RA	19
Insulin	68
Statins	72

**Figure 1:**
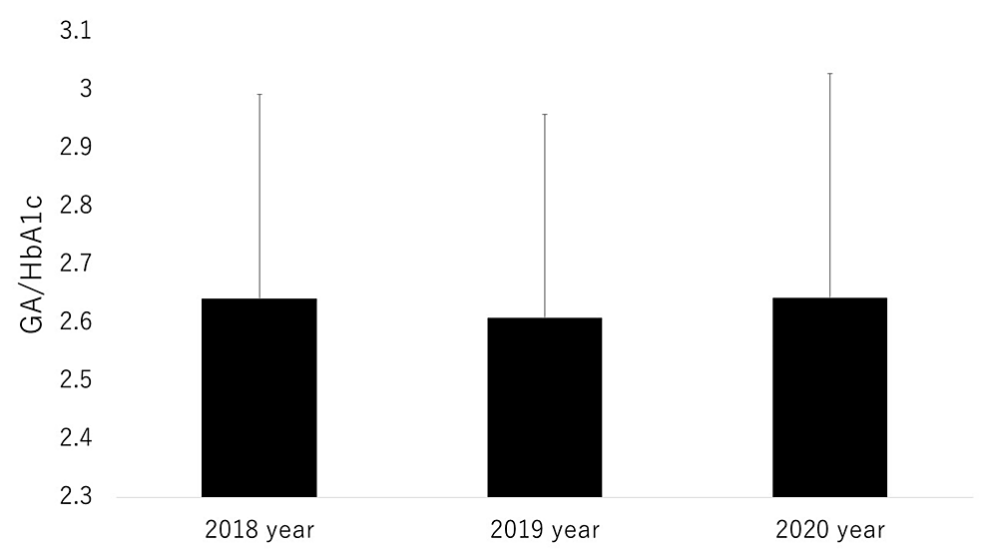
Comparisons of the mean ratios of glycoalbumin (GA) to hemoglobin A1c (HbA1c) (GA/HbA1c) in 2018, 2019, and 2020.

The mean values for GA/HbA1c per year in 2018, 2019, and 2020 were 2.64±0.35, 2.61±0.35, and 2.64±0.39, respectively (n =267 for each year, Figure 1).

There were no significant differences in GA/HbA1c over these years. The differences in HbA1c and GA for 2018, 2019, and 2020 were described in detail in our previous study [29]. In brief, HbA1c and GA were significantly higher in 2020 (COVID-19 pandemic year) compared with 2018 and 2019.

**Figure 2:**
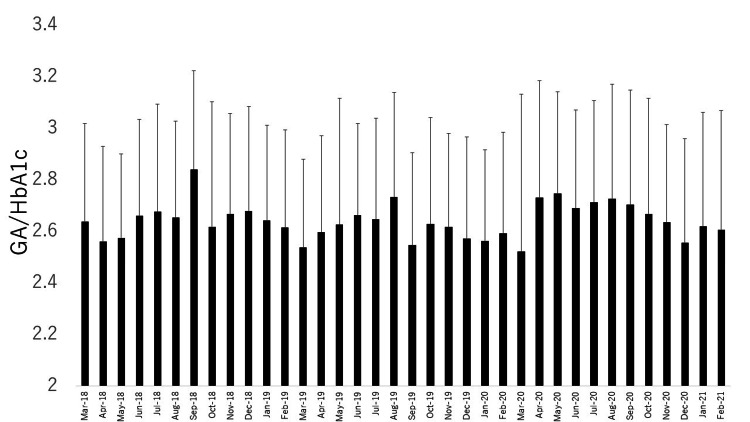
Variations per month over three years for GA/HbA1c. GA/HbA1c: the ratio of glycoalbumin to hemoglobin A1c

**Figure 3:**
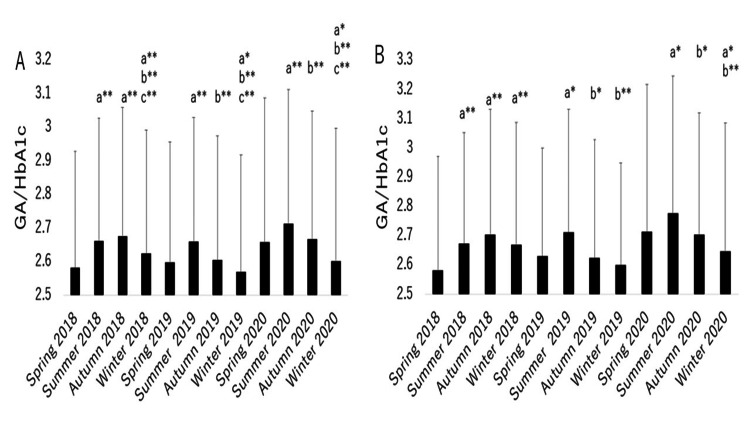
Seasonal variation over three years for GA/HbA1c in all patients (A), and in patients with no change in dose or the type of antidiabetes drugs over the three-year period (B). a: vs. spring; b: vs. summer; c: vs. autumn; **p<0.001; *p<0.05 GA/HbA1c: the ratio of glycoalbumin to hemoglobin A1c

The mean GA/HbA1c values per month and for each season in 2018, 2019, and 2020 are shown in Figure 2 and Figure 3A, respectively.

**Figure 4:**
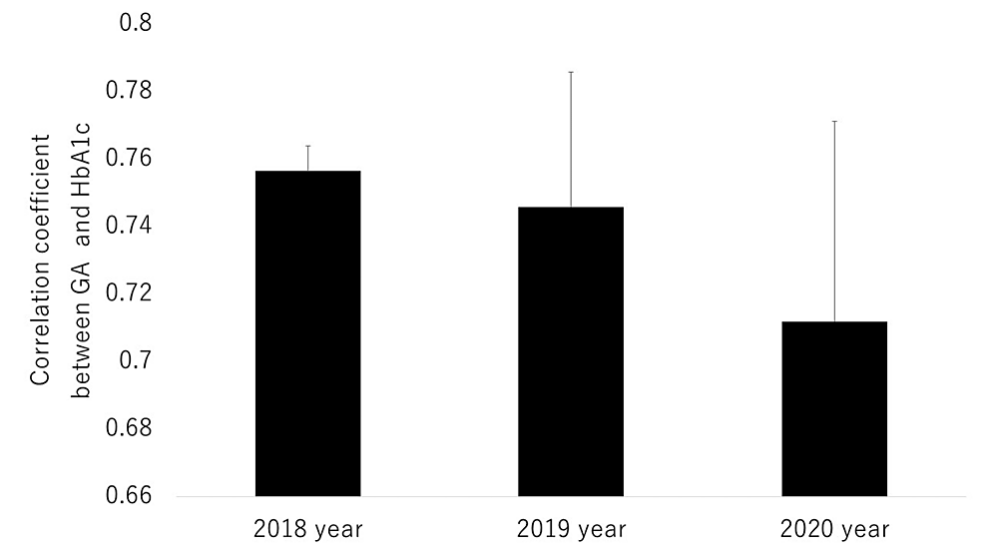
Comparison of mean coefficients between HbA1c and GA in 2018, 2019, and 2020. GA: glycoalbumin; HbA1c: hemoglobin A1c

In all three years, GA/HbA1c was the lowest in winter (2019, 2020) or spring (2018) compared with the other seasons. In addition, in each of the three years, GA/HbA1c in summer was higher than in spring. Therefore, although there was a significant difference in GA/HbA1c between summer and autumn in 2019 and 2020, we concluded that there was a tendency toward a seasonal variation in GA/HbA1c (i.e., higher in summer or autumn and lower in spring or winter). There was a similar pattern for GA/HbA1c in patients in a subgroup (n =85, 2018; n =89, 2019, n =77 in 2020) in which the dose or the type of antidiabetic drugs was not changed, although the tendency appeared to be weaker compared with the overall patient group (Figure 3B). Seasonal variation of HbA1c and GA was investigated in our previous study [29]. In brief, there was an impact of season change (higher in spring and winter, and lower in summer and autumn) on HbA1c each year. On the other hand, there was no apparent impact of season change on GA each year. The mean correlation coefficients between HbA1c and GA in 2018, 2019, and 2020 are shown in Figure 4.

**Figure 5:**
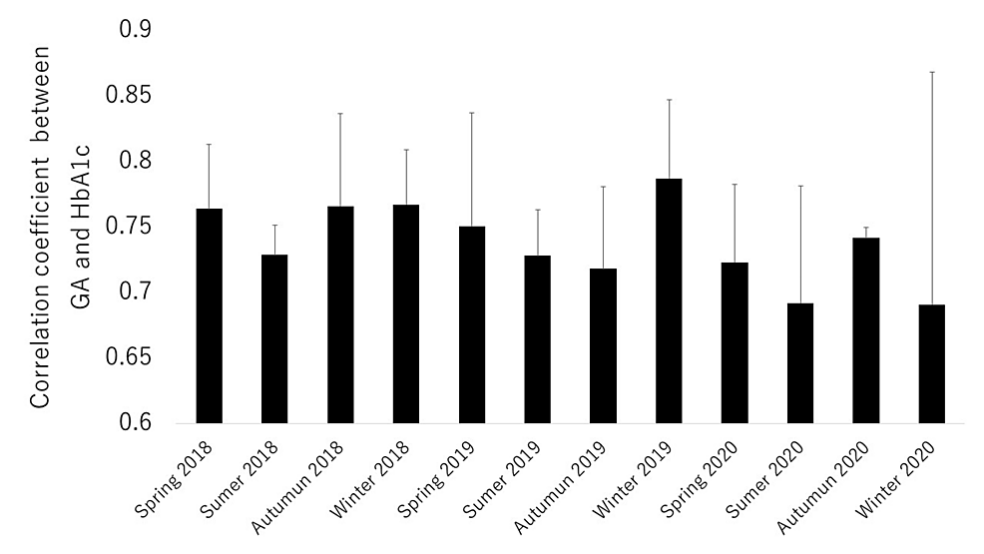
Seasonal variation over three years in the coefficient between HbA1c and GA. GA: glycoalbumin; HbA1c: hemoglobin A1c

There were no differences among these years. The mean correlation coefficients between HbA1c and GA for each season in 2018, 2019, and 2020 are shown in Figure 5.

No significant differences were found among the four seasons in each year. Therefore, we concluded that there was no apparent seasonal variation in this correlation coefficient during these three years.

There were significant positive correlations between the monthly GA/HbA1c and the monthly temperature in Koshigaya City, where our hospital is located, in 2019 (r=0.7253, p=0.0076), and 2020 (r=0.6793, p=0.0372), while no correlation was observed in 2018 (r=0.2340, p=0.4641). The monthly temperature data were obtained from the Japan Meteorological Agency [31].

## Discussion

In the current study, we concluded that GA/HbA1c exhibited a tendency toward seasonal variation (higher in summer or autumn and lower in spring or winter) because GA/HbA1c was the lowest in winter (in 2019 and 2020) or spring (in 2018) compared with the other seasons, and was higher in summer than in spring. An explanation of this seasonal variation of GA/HbA1c is that the seasonal variation of HbA1c itself influenced the ratio, while GA did not show any tendency toward variations throughout the four seasons [29]. Although the reason for the seasonal variation of HbA1c remains unknown, it has been suggested that temperature may influence HbA1c, at least in part [30]. We cannot explain the reason exactly. However, one of the potential reasons might be based on the possible increase of insulin resistance in winter because the tissue sensitivity of glucocorticoids may increase in winter [32]. In our previous study, monthly HbA1c, but not monthly GA, was correlated significantly and negatively with monthly temperature in the city (Koshigaya City) [29]. In the current study, monthly GA/HbA1c similarly showed a significant correlation with monthly temperature in this city in 2019 and 2020, although no correlation was observed in 2018. Therefore, it might be possible that temperature is related to the variation in GA/HbA1c, at least partially; although the involvement appears to be weaker than that for HbA1c.

GA/HbA1c reflects glucose fluctuation or postprandial hyperglycemia [15-18] and is useful as an easily measured marker for these conditions, and therefore as a predictive factor of various complications, including those related to diabetes [19-26]. The results from our study suggest that seasonal variation should be considered when GA/HbA1c is used as a marker for these conditions. In contrast, GA did not show apparent seasonal variation and correlation with temperature, as previously described [29]. The results were similar to a previous report on patients with diabetes [30]. The detailed reason why monthly GA did not show seasonal variations is unclear. However, speculatively, because the seasonal variation of plasma glucose levels is not known, the shorter half-time in the circulation of GA compared with that of HbA1c might be involved at least in part. GA as well as HbA1c could be related to vascular events or mortality in a study with a large number of patients [33] and could predict diabetic complications [34].

Although GA/HbA1c is a good marker of glucose fluctuation or postprandial hyperglycemia [16-19], GA can also reflect glucose fluctuation [13-15]. Therefore, because GA unlike GA/HbA1c or HbA1c is not influenced by seasons, from this point of view, GA might be rather suitable as a stable marker for diabetic complications, including cardiovascular events. On the other hand, we concluded that, although HbA1c and GA themselves were elevated during the COVID-19 pandemic probably due to a decrease in exercise and increased food intake, GA/HbA1c was not influenced by the COVID-19 pandemic because GA/HbA1c did not differ significantly among the three years. This may be an advantage of GA/HbA1c as a marker of diabetic complications. The reason for this result may be partially based on the elevation of both HbA1c and GA during the COVID-19 pandemic. Thus, GA/HbA1c might be a robust marker also in future pandemics. However, the result would have been better supported if the data from 2021 had been included because the effect of the pandemic likely became more expanded in its later years.

In the current study, the strength of the correlations between HbA1c and GA, as evaluated by the correlation coefficients, was similar throughout the three years; the correlation coefficient also did not change during the COVID-19 seasons. In addition, the correlation coefficients did not show any apparent seasonal variation. Therefore, we consider that the strength of the correlation between HbA1c and GA was not influenced by either the COVID-19 pandemic or seasons and that the strength of this correlation is basically constant, except under specific conditions, such as acute changes in glucose levels.

This study was performed in our hospital, located in Koshigaya City. Koshigaya City has four especially distinct seasons and is one of the hottest cities in summer in Japan. Therefore, the difference in temperatures between summer and winter is very large [31]. Thus, the location appears to be suitable to study seasonal variations of HbA1c or GA/HbA1c in patients with diabetes. This is the strength of this study. On the other hand, in the current study, the study design was retrospective and the number of patients investigated was relatively small. The COVID-19 pandemic included only 2020. Although we excluded the patients who had severe anemia, which could influence HbA1 levels, we did not exclude the patients with conditions such as hypothyroidism, liver dysfunction, and malnutrition, which can influence GA levels [35]. These are considered the limitations of this study.

## Conclusions

In patients with type 2 diabetes, GA/HbA1c had a tendency toward showing seasonal variation, which was not influenced by the COVID-19 pandemic. It may be necessary to consider seasonal variation when GA/HbA1c is used as a marker of glucose fluctuation or as a predictor of various complications. On the other hand, GA/HbA1c did not differ significantly among the three years, including 2020 (i.e., a COVID-19 pandemic year). Thus, GA/HbA1c might be useful as a robust marker of diabetic complications in future pandemics. Furthermore, the strength of the correlations between HbA1c and GA, as evaluated by the correlation coefficients, was similar throughout the three years, and the correlation coefficients did not show any apparent seasonal variation. Therefore, the strength of the correlation between HbA1c and GA may not be influenced by either pandemics such as COVID-19 or seasons.
